# Telehealth: a new opportunity for out-patient psychiatric services

**DOI:** 10.1192/bji.2022.34

**Published:** 2023-05

**Authors:** Erica Bell, Cornelia Kaufmann, Gin S. Malhi

**Affiliations:** 1 Mood-T Coordinator and Scientific Officer, CADE Clinic and Mood-T Service, Mental Health Drug and Alcohol, Northern Sydney Local Health District, Academic Department of Psychiatry, Kolling Institute, Northern Clinical School, Faculty of Medicine and Health, The University of Sydney, New South Wales, Australia. Email erica.bell@sydney.edu.au; 2 Lecturer, CADE Clinic and Mood-T Service, Mental Health Drug and Alcohol, Northern Sydney Local Health District, Academic Department of Psychiatry, Kolling Institute, Northern Clinical School, Faculty of Medicine and Health, The University of Sydney, New South Wales, Australia; 3 Professor, CADE Clinic and Mood-T Service, Mental Health Drug and Alcohol, Northern Sydney Local Health District, Academic Department of Psychiatry, Kolling Institute, Northern Clinical School, Faculty of Medicine and Health, The University of Sydney, New South Wales, Australia

**Keywords:** Telehealth, clinical psychiatry, bipolar affective disorders, depressive disorders, psychosocial interventions

## Abstract

In the wake of the COVID-19 pandemic, healthcare systems rapidly embraced technology as a means of providing care while adhering to social distancing protocols. In this brief article, we report on a new telehealth initiative recently implemented in an out-patient psychiatric setting and outline the novel role telehealth may serve in facilitating psychiatric care globally. The uptake of telehealth represents a new and exciting opportunity to increase both access to, and quality of, care for people with mental illness.

Globally, we recognise that more needs to be done to improve access to care for psychiatric patients. Individuals with psychiatric disorders are often not able to avail themselves of the care they need, and even those that do receive care experience difficulties.

The recent COVID-19 global pandemic illustrated how fragile healthcare systems are to additional stressors and showed that when problems do arise, primary care and psychiatric care are often the services that are most heavily affected.^1^ For instance, assessments are not comprehensive and follow-up is either too infrequent or absent altogether.^2^ It is difficult to find a silver lining to this particular cloud, but one positive outcome of the restrictions to movement caused by the pandemic is the realisation of the potential for technology to improve both access to care and the quality of care for out-patients. Not only do we have the capability to utilise technology to do this, but it has become highly acceptable to both patients and clinicians as a means of accessing care.^3^

Of course, telehealth cannot completely replace face-to-face care, but it is a perfectly adequate substitute in many instances and at the very least can serve as a key facilitator. For example, in out-patient psychiatric care, it can work synergistically alongside face-to-face interactions with a clinician (general practitioner, psychiatrist or psychologist). Therefore, in this article we report on a new initiative utilising telehealth in a specialist out-patient service in Sydney (Australia) that has been instituted recently.

## The need for telehealth

Mood disorders, such as depression and bipolar disorder, are common and confer a high burden of disease and disability.^4^ This is because they are recurrent and chronic illnesses that usually commence early in life and often require specialised care. In line with the current recommended best practices for the management of mood disorders,^5^ this entails a longitudinal treatment plan to prevent presentations to hospital and to minimise impairment in functioning, and such care should involve regular communication and coordination between all clinicians involved in the treatment of the patient. Furthermore, successful management requires regular review and re-evaluation of treatment response and appraisal of patient outcomes.

Telehealth makes it possible for more patients to access specialist mood disorder services. These are usually only available in specialist centres, which are typically situated in large towns and cities, especially if affiliated with universities. Specialist services often involve sophisticated review of diagnosis and more detailed management formulation, which often includes follow-up evaluations to longitudinally assess the effect of treatments that have been trialled. Such specialist centres are also able to provide a consistent point of communication between the patient and clinicians involved in their care. It is here that telehealth is able to facilitate the systematic, coordinated care between a network of professionals, in line with the recommended best practices for the management of mood disorders. Indeed, this aspect is perhaps better achieved in telehealth than by conventional means.

For example, recent guidelines for the management of depression advocate lifestyle changes, psychoeducation and the cessation of smoking, alcohol or drug use as first-line interventions,^5,6^ and this advice can be readily implemented via telehealth sessions conducted by allied health staff (nurses, dieticians, occupational therapists, physiotherapists). This lays the foundation for psychological interventions and engagement with mental health services. In practice, most patients with mood disorders will need to trial a number of treatments, including both psychological and pharmacological, before they experience a significant and sustained improvement in symptoms that leads to a satisfactory recovery.^7^ This fine-tuning of treatment requires cohesion and communication between members of a treatment team. It is with these broad aims in mind and because of the constraints of the pandemic that we decided to develop an existing clinical out-patient service and add a telehealth facility.

## The Mood-T service

**Fig. 1:**
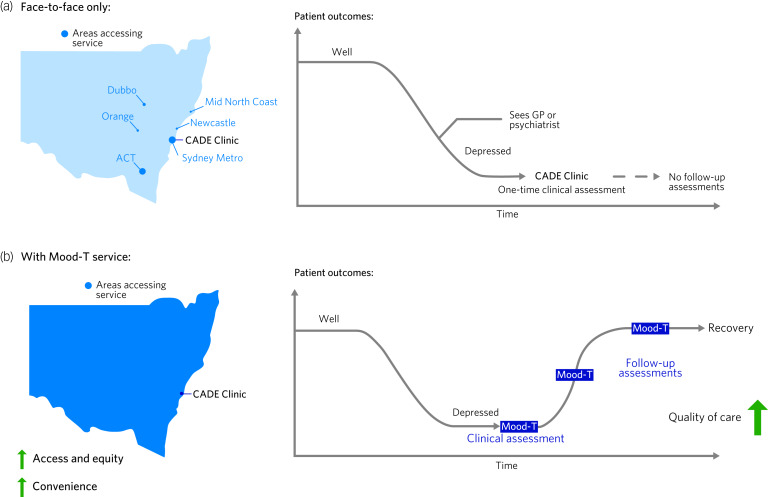
Improved access and quality of care utilising a telehealth model. (a) Both the access and quality of care provided is limited in a face-to-face only model. Those accessing the service face-to-face largely came from areas close to Sydney or from larger regional towns, which nevertheless required significant travel times (Dubbo and the Australian Capital Territory (ACT) are 5 and 3 hours’ drive from Sydney respectively). Furthermore, although patients attended an initial assessment, it was impractical to ask them to return for a re-evaluation or follow-up assessment. (b) Following the implementation of the telehealth service (Mood-T), we are now able to assess patients from anywhere within the state (enhanced access and equity) and can organise follow-up assessments at any time (enhanced convenience). Furthermore, other clinicians involved in the patient's care can be invited to join the online consultation to take part in the assessment.

The Mood Disorders Telehealth (Mood-T) service recently launched in Sydney allows for enhanced access and quality of care for individuals across the state of New South Wales (Fig. 1). Because this state is relatively large (>800 000 km^2^) it is difficult for individuals who live outside of metropolitan Sydney to access specialist care for complex mood disorders. Furthermore, even if individuals are able to access care it may be difficult and time-consuming to coordinate their care between multiple clinicians, especially if they cannot meet and contribute to management decisions within the consultation, as is often the case. Telehealth addresses both of these barriers. However, it is important to note that although the Mood-T service offers assessments and follow-up, it does not assume primary responsibility for patient care. Instead, it provides diagnosis and management advice to the existing network of clinician(s) involved in the care of the patient and provides a node that further extends the network of care.

## Operationalising a telehealth service

**Fig. 2:**
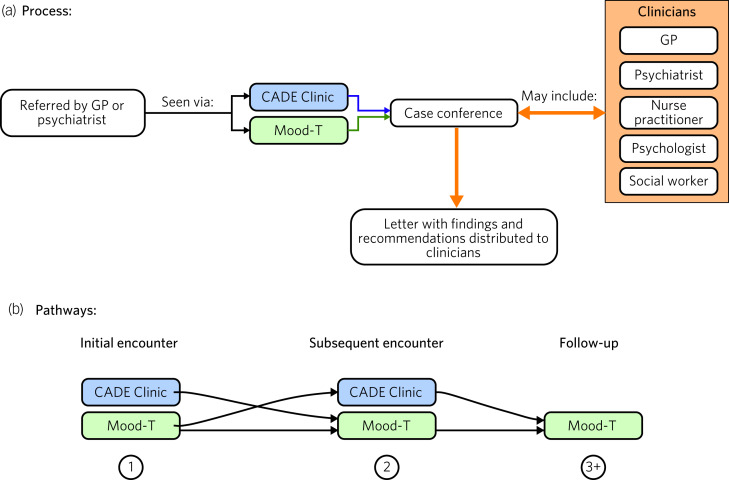
Process and pathways utilised in the Mood-T service. (a) The components of a typical assessment, including the various clinicians who may be involved as required. (b) The pathways for our specific service, wherein patients may be seen via any sequential combination of telehealth and in-person appointments where appropriate and necessary. For example, the initial encounter (1) and the subsequent encounter (2) may be conducted either in-person or via telehealth, and follow-up (3) may be conducted virtually. These are the approaches we have utilised in our service, and naturally these pathways of patient flow may be adapted to suit new telehealth services with different local circumstances and resources.

The Mood-T service provides a virtual platform for our specialist mood disorders out-patient service (the CADE Clinic, www.cadeclinic.com) and extends its reach considerably. This service receives referrals largely from general practitioners and psychiatrists and offers detailed clinical assessments of patients with mood disorders. These assessments are conducted with the aim of providing clarity regarding diagnosis and management. In all assessments, patients usually complete a battery of clinical questionnaires before attending the appointment; they are then seen by several members of the clinical team sequentially. Following this, the team have a case conference to discuss the diagnosis and management plan, and finally a meeting involving the entire clinical team and the patient is held to relay the findings of the assessments to the patient. A letter detailing the outcomes of the assessment is then sent to the referring clinician, and all other relevant clinicians involved in the care of the patient are informed (Fig. 2).

## Advantages to implementing a telehealth model

As noted above, virtual assessments are not the same as being in the same room as a patient, and there are some limitations to conducting assessments virtually. For example, you cannot conduct a physical examination, and if there is more than one person in the room then interacting with a group via telehealth can sometimes be difficult. However, there are also several advantages to implementing a telehealth model, and these are outlined in Box 1.

Box 1 Advantages and benefits of implementing a telehealth service

Security and confidentiality

- The security and privacy of a telehealth service can now be guaranteed (e.g. Mood-T is facilitated through the local health district's private and secure server).

Familiarity

- Patients and clinicians are now highly computer literate and are often experienced in using videoconferencing software, and almost all patients can access at least one compatible device (e.g. smartphone, tablet, computer).

Flexibility

- It allows for flexibility, as clinicians and carers can be added to and removed from the virtual consultation room as needed.

Versatility

- Important psychoeducation materials or notes can be digitally shared with the patient in real time.
- Enhanced versatility, as one can switch between virtual locations and rooms easily and quickly.

Convenience

- Patients can attend the assessment from a convenient and private location and device.
- It is not limited by concerns about infections (e.g. influenza, COVID-19, the common cold), as there is no physical contact.

Efficiency and access

- Telehealth allows for more immediate appointments to be made with little notice (as neither the patient or the clinician need to travel to attend).
- Telehealth allows access for those with limited mobility, independence or other barriers to attending in-person appointments.
- Telehealth removes potentially significant financial and time costs of attending in-person appointments, e.g. patients do not have to take time off work, seek childcare, pay for travel expenses.

## Conclusions

In summary, telehealth offers a significant opportunity for improving both access to care and quality of care for psychiatric patients. In our particular service, which provides assessments and management formulations for out-patients with mood disorders, telehealth has significantly expanded our reach and has facilitated long-term follow-up of patients, especially those living in rural or remote areas. This has enabled us to assess whether the treatment recommendations have been implemented, whether they have been successful in achieving a response and whether there have been any barriers faced by patients, which we can then learn from and accommodate. Thus, we feel that the advantages that telehealth confers should be utilised where possible in existing psychiatric services, in tandem with in-person contact.

Given the immense need for psychiatric services and the cost-efficiency of this means of delivery, we feel this is the kind of model of care that needs to be adopted more widely, especially in areas of need. And although Australia is a high-income country there are still significant disparities in socioeconomic status between metropolitan and rural/remote areas for example. Similarly, in other parts of the world, where psychiatric services are scarce, establishing telehealth services may allow greater access and enable the provision of cost-effective care.

## Data Availability

Data availability is not applicable to this article as no new data were created or analysed in this study.
